# A Metagenome-Wide Association Study of the Gut Microbiome and Metabolic Syndrome

**DOI:** 10.3389/fmicb.2021.682721

**Published:** 2021-07-16

**Authors:** Qian Qin, Su Yan, Yang Yang, Jingfeng Chen, Tiantian Li, Xinxin Gao, Hang Yan, Youxiang Wang, Jiao Wang, Shoujun Wang, Suying Ding

**Affiliations:** ^1^Health Management Center, The First Affiliated Hospital of Zhengzhou University, Zhengzhou, China; ^2^College of Public Health, Zhengzhou University, Zhengzhou, China; ^3^Department of Geriatric Endocrinology, The First Affiliated Hospital of Zhengzhou University, Zhengzhou, China; ^4^Department of Endocrinology, The First Affiliated Hospital of Zhengzhou University, Zhengzhou, China

**Keywords:** metabolic syndrome, gut microbiota, metagenomics, comparative genomics, metabolic pathway

## Abstract

Metabolic syndrome (MetS) is a wide-ranging disorder, which includes insulin resistance, altered glucose and lipid metabolism, and increased blood pressure and visceral obesity. MetS symptoms combine to result in a significant increase in cardiovascular risk. It is therefore critical to treat MetS in the early stages of the disorder. In this study, 123 MetS patients and 304 controls were recruited to determine whether the gut microbiome plays a role in MetS development and progression. By using whole-genome shotgun sequencing, we found that the gut microbiomes of MetS patients were different from those of controls, with MetS patients possessing significantly lower gut microbiome diversity. In addition, 28 bacterial species were negatively correlated with waist circumstance, with *Alistipes onderdonkii* showing the strongest correlation, followed by *Bacteroides thetaiotaomicron*, *Clostridium asparagiforme*, *Clostridium citroniae*, *Clostridium scindens*, and *Roseburia intestinalis*. These species were also enriched in controls relative to MetS patients. In addition, pathways involved in the biosynthesis of carbohydrates, fatty acids, and lipids were enriched in the MetS group, indicating that microbial functions related to fermentation may play a role in MetS. We also found that microbiome changes in MetS patients may aggravate inflammation and contribute to MetS diseases by inhibiting the production of short-chain fatty acids (SCFAs). Taken together, these results indicate the potential utility of beneficial gut microbiota as a potential therapeutic to alleviate MetS.

## Introduction

Metabolic syndrome (MetS) is defined by the International Diabetes Federation (IDF) based on central obesity, as determined by waist circumference (WC; Alberti et al., 2005). MetS pathophysiology involves insulin resistance and fat metabolism disorders resulting from visceral obesity. A study in 2014–2015 showed that 107 million Chinese adults aged 40–74 had been diagnosed with MetS, according to the adult treatment panel III (ATP III) definition (Li et al., 2018), which results in an increased risk of diabetes mellitus (Hudish et al., 2019), hypertension (Farmer, 2004), and morbidity and mortality from cardiovascular disease (Bloomgarden, 2007; Sakurai et al., 2010). These secondary disorders that result from MetS bring with them heavy medical and economic burdens to individual patients and society as a whole, highlighting the urgent need to reduce the risk factors associated with MetS.

In addition to genetic factors, differences in diet and lifestyle have been linked to unhealthy microbiomes, resulting in increased inflammation and a higher risk of MetS (Hills et al., 2019; Gildner, 2020). Previous studies have shown that a higher Firmicutes-to-Bacteroidetes ratio increased obesity prevalence in mice fed high-fat diets (HFDs; Hills et al., 2019). In addition, a multi-ethnic population study showed that abundances of the Peptostreptococcaceae and Turicibacter families decreased, while Enterobacteriaceae increased in MetS patients (Deschasaux et al., 2020). These studies indicate that the microbiome of obese individuals may be more efficient at harvesting energy from diet, which, in turn, could lead to higher body mass index (BMI) and insulin resistance (Mazidi et al., 2016).

In addition, intestinal microbes may lead to obesity by affecting the metabolism of other symbiotic bacteria and causing chronic inflammation from short-chain fatty acids (SCFAs), which are the fermentation product of polysaccharides. Polysaccharides have been shown to positively regulate the microbiota population structure by promoting the proliferation of beneficial bacteria and inhibiting the proliferation of harmful bacteria (Tao et al., 2017). Polysaccharides can upregulate enzymes involved in carbohydrate metabolism (CAZymes). This increases the production of SCFAs, which enhance the health of the intestinal barrier by promoting mucus production and connexin expression, reducing metabolic endotoxins and expression of inflammatory factors (Bell and Juge, 2020). A previous study found that the positive effects of *Lactobacillus plantarum* PMO 08 on MetS were related to microbiota modulation and intestinal barrier integrity in obese mice fed a HFD, with the primary effect coming through the regulation of lipid metabolism (Oh et al., 2019). Furthermore, the steroidal glycoside ophiopogonin D (OP-D) alleviates MS and insulin resistance in male mice fed a HFD by decreasing the Firmicutes-to-Bacteroidetes ratio and endotoxin-bearing Proteobacteria levels, which indicates that polysaccharides may be used as a prebiotic agent to treat obesity-associated gut dysbiosis and MetS (Chen et al., 2018). In addition, a significant improvement in insulin sensitivity in conjunction with increased intestinal microbial diversity, including a distinct increase in butyrate-producing bacterial strains, were found in men with MetS after lean male donor fecal microbiota transplantation (Hartstra et al., 2015; Li et al., 2018). Products of intestinal microbes, such as butyrate, may induce beneficial metabolic effects through enhancement of mitochondrial activity, prevention of metabolic endotoxemia, and activation of intestinal gluconeogenesis *via* gene expression and hormone regulation.

Based on the role that SCFAs play in obesity and the differences in gut microbiota composition in MetS subjects in a multi-ethnic population, this study aims to reveal how the metabolites of the intestinal microbiota act on MetS in order to provide a theoretical basis for the treatment of MetS by analyzing characteristics of the intestinal microbiota of MetS patients in China.

## Materials and Methods

### Study Population

For this study, 290 men and 137 women from the First Affiliated Hospital of Zhengzhou University were selected from 2018 to 2019 to participate in the study after volunteering for a physical examination. Among these, 31 women and 92 men were diagnosed with MetS based on the IDF definition and were aged 44.82 ± 11.86 years, and 106 women and 198 men were in the control group and aged 42.93 ± 11.38 years. All studies involving human participants were reviewed and approved by an ethics committee from the First Affiliated Hospital of Zhengzhou University.

### Data Collection

A questionnaire and physical examination were conducted by trained staff in the First Affiliated Hospital of Zhengzhou University, to collect data on demographic characteristics, medical history, behavioral risk factors, height, weight, WC, and blood pressure. Subjects were required to have blood drawn after 12 h of fasting and serum testing results of white blood cells (WBC), neutrophil count (NEC), high-density lipoprotein cholesterol (HDL), low-density lipoprotein cholesterol (LDL), triglycerides (TG), total cholesterol (TC), fasting blood glucose (FBG), glycated hemoglobin A1c (HbA1c), and uric (UC) of the subjects were obtained through the hospital laboratories. Additionally, quantification computed tomography (QCT PRO V6.1, Mindways, United States) was used to detect visceral fat area (VFA). On the same day, fecal samples were collected and separately packed and then placed in a −80°C refrigerator for later testing.

### DNA Extraction, Shotgun Metagenomic Sequencing, and Quantity Control of Reads

According to the manufacturer’s instructions, DNA was extracted from a total of 1,770 stool samples using the MagPure Stool DNA KF kit. DNA library construction based on DNA nanospheres (DNB) and shotgun metagenomic sequencing based on combined probe anchoring synthesis (CPAS) were performed on all samples (MGI2000, MGI, Shenzhen, China). The overall accuracy (OA ≥ 0.8) control strategy described above was used to carry out quality control (QC) on original sequenced reads to filter out low-quality reads.

### Microbiome Composition and Function Profiling

Taxonomic annotation and quantification were performed based on MetaPhlAn2 with default settings, generating gut microbial profiling that included bacteria, archaea, eukaryotes, and viruses. Firstly, compare sequencing readings with classification markers. The MetaPhlAn2 classifier compares metagenomic reads against this precomputed marker catalog using nucleotide BLAST searches to provide clade abundances. Then, calculation of intestinal microflora content. The classifier normalized the total number of reads in each clade by the nucleotide length of its markers, taking into account any markers specific to subclades. Taxon-specific community functional profiles were further generated using HUMAnN2 (the HMP Unified Metabolic Analysis Network 2).

### Statistical Analysis

Statistical analyses were performed using the R program, version 4.0.2. Standardized statistical test methods were used to analyze the results of demography and laboratory tests. Categorical variables were represented by count, and chi-square tests were used for association testing. Continuous variables were expressed as means ± SD. The analysis of differences between groups was performed by a normality test and a homogeneity test; *p*-value ≥ 0.05 was considered to be normal and homogeneous, followed by parametric testing or nonparametric testing; *p*-value *<* 0.05 was considered to be statistically significant. We used the R package “vegan” to calculate the Shannon index and Gini index and the Hellinger, Bray, JSD, and Pearson indices of each sample. Principal coordinate analysis (PCoA) was performed by the R program “ade4” to perform visual analysis. The Wilcoxon test was used to analyze the differences between groups of microbiota and pathways, and *p* < 0.05 was considered statistically significant. Spearman correlation analysis was used to analyze the correlation between differential flora and covariates, and the “corrplot” package was used for visualization.

## Results

### Clinical Characteristics of Subjects

A total of 137 women and 290 men were enrolled in this study. Among them, 123 were diagnosed with MetS and 304 were in the control group, which had fewer than two MetS-associated markers. There were no statistically significant differences in the age or gender composition of the two groups. The parameters WC, BMI, VAF, WBC, and NEC were significantly (*p* < 0.05) higher in the MetS group compared with the control group, but no major difference was seen for the other indicators (Table 1).

**TABLE 1:** The major demographic and serum characteristics of the MetS subjects and the control group.

	MetS (*n* = 123)	Control (*n* = 304)	*p*
Gender	Female: 31, male: 92	Female: 106, male: 198	0.068
Age	44.82 ± 11.86	42.93 ± 11.38	0.134
WC	93.79 ± 11.75	89.66 ± 9.93	0.001*
BMI	26.24 ± 3.93	24.80 ± 3.26	0.000*
VFA	104.26 ± 36.56	94.03 ± 32.29	0.007*
SBP	139.43 ± 13.74	124.31 ± 15.25	0.000*
DBP	88.01 ± 8.95	75.11 ± 10.84	0.000*
FBG	5.60 ± 1.44	5.32 ± 1.37	0.065
HbA1c	5.95 ± 0.86	5.81 ± 0.80	0.196
TC	4.75 ± 0.79	4.70 ± 0.90	0.562
TG	1.86 ± 1.29	1.61 ± 1.12	0.068
HDL	1.34 ± 0.40	1.37 ± 0.36	0.519
LDL	2.89 ± 0.74	2.88 ± 0.79	0.832
UC	342.35 ± 83.66	326.33 ± 87.67	0.079
WBC	6.49 ± 1.72	6.05 ± 1.55	0.015*
NEC	3.84 ± 1.30	3.56 ± 1.14	0.037*

WC, waist circumference; BMI, body mass index; VFA, visceral fat area; SBP, systolic blood pressure; DBP, diastolic blood pressure; FBG, fasting blood glucose; HbA1c, glycated hemoglobin A1c; TC, total cholesterol; TG, triglyceride; HDL, high-density lipoprotein; LDL, low-density lipoprotein; UC, uric; WBC, white blood cell; NEC, neutrophil count. Differences between MetS subjects (*n* = 123) and the control group (*n* = 304) were compared by using Student’s *t*-tests for normal continuous variables. Statistical significance was defined as *p* < 0.05. *p < 0.05.

### Analysis of Microbiota Diversity

**FIGURE 1:**
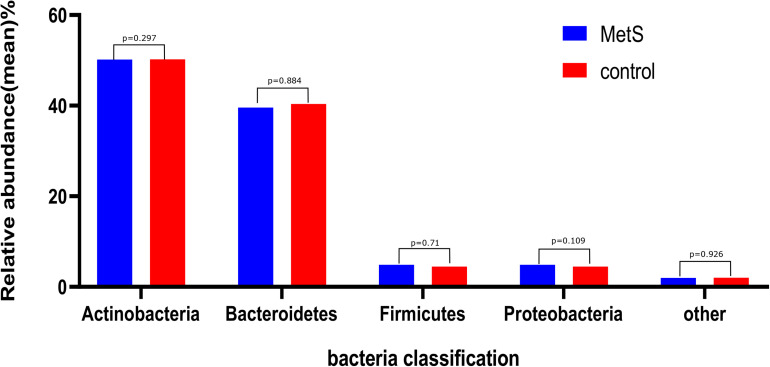
The gut microbiota composition of the MetS and control groups. In both the MetS and control groups, gut microbiota was dominated by four abundant phyla, namely, Bacteroidetes (50.22 and 50.15%), Firmicutes (40.40 and 39.60%), Proteobacteria (4.50 and 4.88%), and Actinobacteria (2.32 and 2.19%). There were no significant differences in the phyla of the MetS compared to the control group (*p* > 0.05).

We sought to classify the identified sequences into phyla and genera based on their closest match in the reference database. Overall, gut microbiota was dominated by four abundant phyla, which were present at very similar levels in the MetS and control groups: Bacteroidetes (50.22 and 50.15%), Firmicutes (40.40 and 39.60%), Proteobacteria (4.50 and 4.88%), and Actinobacteria (2.32 and 2.19%) (Figure 1).

**FIGURE 2:**
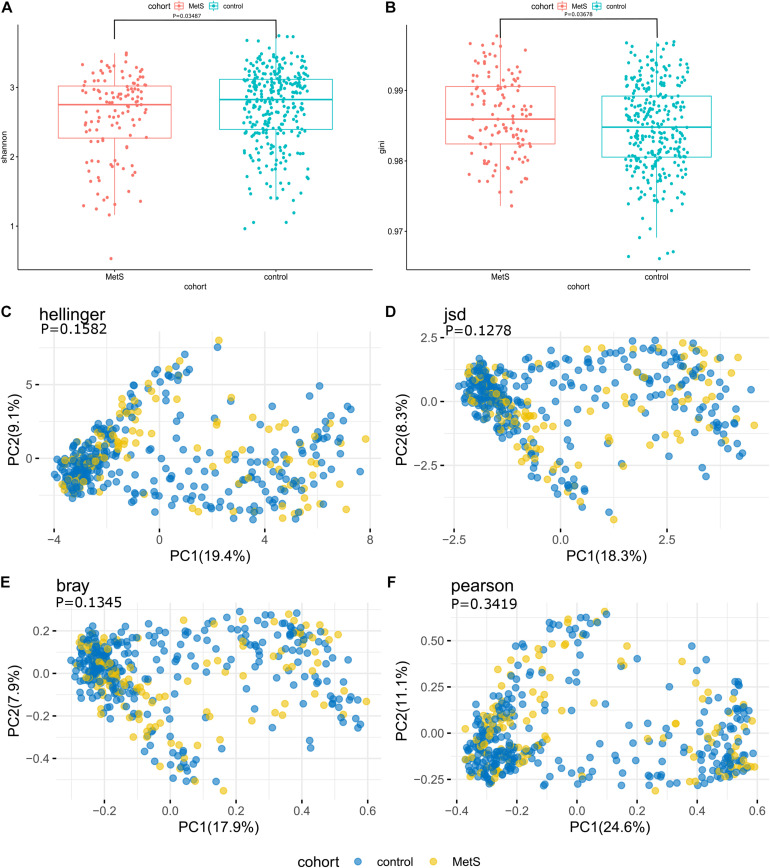
Alpha diversity, beta diversity, and bacterial profiles of feces. **(A,B)** Alpha diversity was measured by both Shannon index and Gini index for comparisons between the MetS and control groups. The MetS subject microbiomes had significantly lower species-level alpha diversity compared to the microbiomes of the control group (*p* = 0.03487 and *p* = 0.03678). **(C–F)** Beta diversity between the MetS group and control group. Beta diversity was calculated based on the Hellinger distance, JSD distance, Bray distance, and Spearman distance, and no significant differences were found between the MetS and control groups.

At the species level, the microbiomes of the MetS subjects had significantly lower alpha diversity when compared with the microbiomes of the control subjects, as measured by both Shannon index and Gini index (*p* = 0.03487 and *p* = 0.03678) (Figures 2A,B). To compare the species-level beta diversity of the MetS and control groups, the Hellinger distance, Jensen-Shannon divergence (JSD) distance, Bray distance, and Spearman distance were calculated. This analysis revealed that there was no significant difference in the species-level beta diversity between the MetS and control groups (*p* = 0.1582, *p* = 0.1278, *p* = 0.1345, and *p* = 0.3419, respectively, Figures 2C–F).

### Analysis of Microbiota Composition and Association Among the Gut Microbiomes and Clinical Characteristics

**FIGURE 3:**
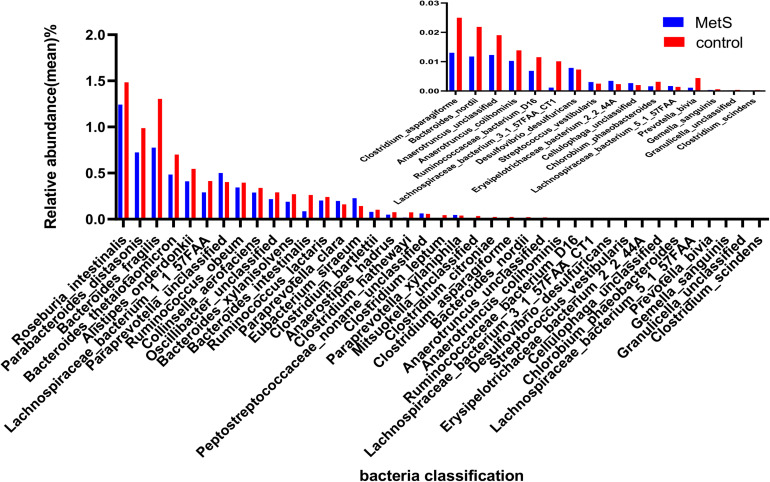
The relative abundance of bacterial species between the MetS and control groups. We used Wilcoxon tests to analyze the relative abundance of bacterial species and found significant differences in 39 species, with 31 enriched in the control group and 8 enriched in the MetS group. *p* < 0.05.

Our species-level analysis found that 71 species had significantly different abundances in the MetS group versus the control group. After removing the species with low occurrence and abundance, 39 species with significantly different abundance remained (*p* < 0.05; Figure 3), 31 of which were enriched in the control group and 8 of which were enriched in the MetS subjects. Wilcoxon tests showed that the relative abundance of Clostridiales (including *Chlorobium phaeobacteroides*, *Clostridium asparagiforme*, *Clostridium bartlettii*, *Clostridium leptum*, *Clostridium scindens*, and *Collinsella aerofaciens*) was enriched in controls. Five species belonging to the order Bacteroidales (*Bacteroides fragilis*, *Roseburia intestinalis*, *Bacteroides nordii*, *Bacteroides thetaiotaomicron*, and *Bacteroides xylanisolven*), four species belonging to the genus *Alistipes* (*Alistipes onderdonkii*, *Alistipes hadrus*, *Alistipes colihominis*, and unclassified), and three species belonging to the family Ruminococcaceae (bacterium D16, *Ruminococcus lactaris*, and *Ruminococcus obeum*) were enriched in controls. *Paraprevotella xylaniphila*, unclassified *Paraprevotella*, *Streptococcus vestibularis*, unclassified *Cellulophaga*, *Eubacterium siraeum*, *Paraprevotella clara*, *Gemella sanguinis*, and unclassified Peptostreptococcaceae were found to be enriched in the MetS subjects.

**FIGURE 4:**
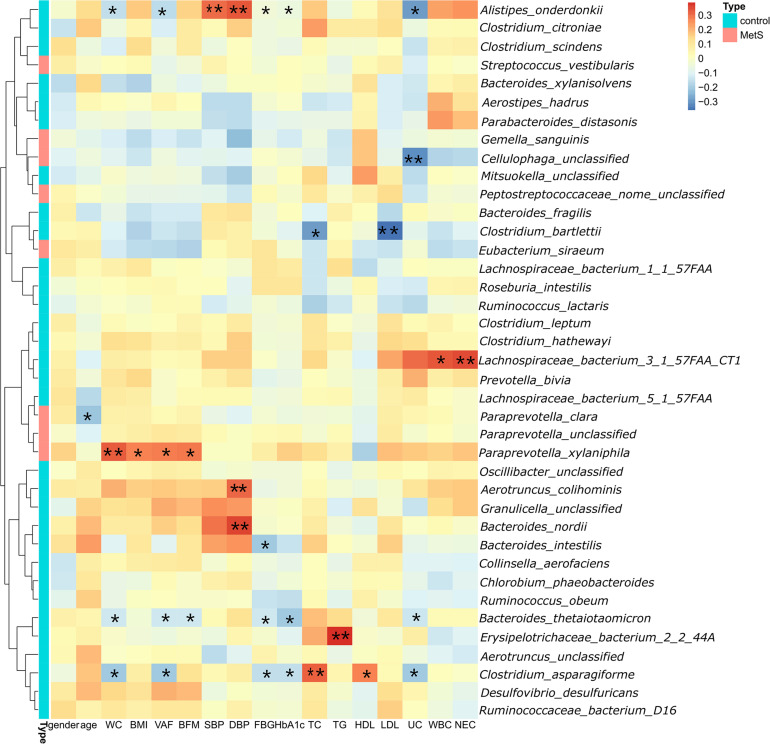
Significant correlations between bacteria and clinical characteristics. We found 28 species in which the abundance was negatively correlated with WC. Three species (*Alistipes onderdonkii*, *Bacteroides thetaiotaomicron*, and *Clostridium asparagiforme*) were negatively correlated with WC, VFA, FBG, HbA1c, and UC.*p < 0.05, **p < 0.01.

Spearman’s correlation analysis was used to explore the correlations between species abundances and the clinical characteristics. We found 28 species with abundances that were negatively correlated with WC (Figure 4), with *A. onderdonkii* abundance showing the strongest correlation, followed by *B. thetaiotaomicron, C. asparagiforme, Clostridium citroniae, C. scindens*, and *R. intestinalis*. Three species (*A. onderdonkii, B. thetaiotaomicron*, and *C. asparagiforme*) were negatively correlated with WC, VFA, FBG, HbA1c, and UC.

### Functional Shifts in the Microbiome Characteristics of Different Subjects

**FIGURE 5:**
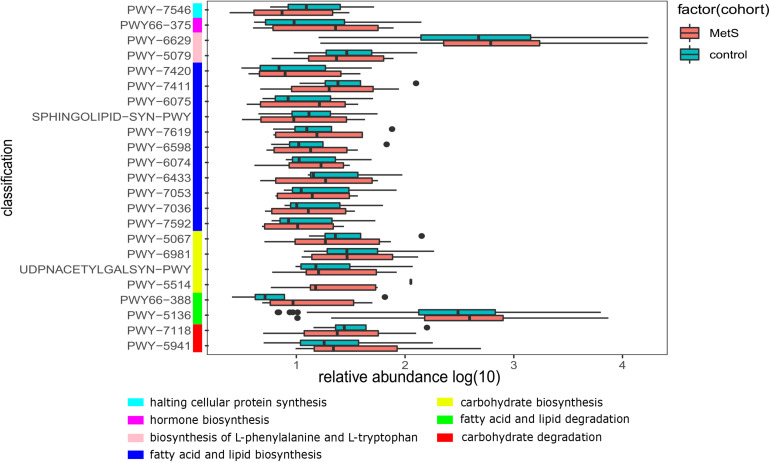
The functional shifts of bacterial species between the MetS and control groups. We found that 64 pathways were significantly different, and 57 pathways were enriched in MetS subjects. *p* < 0.05. Within the 57 MetS-enriched pathways, we showed the 23 pathways that were responsible for metabolism of carbohydrates, fatty acids, lipids, amino acid, hormones, and halting cellular protein synthesis.

We also constructed functional profiles for each sample using 494 microbial MetaCyc pathways. After removing low abundance pathways, a total of 64 MetaCyc pathways were found to be significantly different when comparing the MetS and control groups (Figure 5), 57 of which were enriched in MetS subjects. Within the 57 MetS-enriched pathways, 4 were responsible for degradation (carbohydrate degradation: PWY-5941 and PWY-7118 and fatty acid and lipid degradation: PWY-5136 and PWY66-388), 4 pathways (PWY-5514, UDPNACETYLGALSYN-PWY, PWY-6981, and PWY-5067) were responsible for carbohydrate biosynthesis, and 11 pathways (PWY-7592, PWY-7036, PWY-7053, PWY-6433, PWY-6074, PWY-6598, PWY-7619, SPHINGOLIPID-SYN-PWY, PWY-6075, PWY-7411, and PWY-7420) were responsible for fatty acid and lipid biosynthesis. Two pathways enriched in the MetS group (PWY-5079 and PWY-6629) were involved in the biosynthesis of L-phenylalanine and L-tryptophan. In addition, the PWY66-375 pathway is involved in hormone biosynthesis. Other pathways enriched in MetS subjects were responsible for macromolecule modification, electron carriers, and vitamins such as folic acid. Pathways enriched in MetS subjects were involved in degradation and biosynthesis of carbohydrates, fatty acids, and lipids, indicating that the microbial functions of fermentation and utilization of carbohydrates, fatty acids, and lipids were closely related to MetS.

## Discussion

There is increasing evidence that recent changes in lifestyles have led to a rapid increase in metabolic disorders, including insulin resistance, alterations in glucose and lipid metabolism, increased blood pressure, and visceral obesity. All of these changes combine to result in a significant increase in cardiovascular risk (Bozkurt et al., 2016). At present, the treatments for metabolic disorders mainly depend on pharmaceutical interventions and lifestyle changes. However, recent research on gut microbes and obesity-related diseases has provided a new area to investigate for the treatment of MetS. In view of the differences found in gut microbes among different ethnic populations, this article analyzes the characteristics of gut microbes and microbial metabolic pathways in the Han nationality in the Chinese population with MetS, providing a new avenue to explore for the treatment of MetS.

As defined by the IDF, MetS is primarily based on central obesity reflected by a high WC. Our metagenome-wide association study found that among the MetS parameters, WC had the largest number of correlations with gut microbial species. Few studies have explored the relationships between gut microbiota and visceral fat. Nie et al. (2020) found that members of the families Clostridiales and Lachnospiraceae were strongly negatively correlated with obesity parameters such as WC, VFA, TG, LDL, HbA1c, SBP, and DBP. Most of these correlations were negative, which supports the findings of our analysis. In order to prevent false positives, bacterial species were required to appear in at least 10 samples. We found that six representative species (*A. onderdonkii*, *B. thetaiotaomicron*, *C. asparagiforme*, *C. citroniae*, *C. scindens*, and *R. intestinalis*) were enriched in healthy controls, which were negatively correlated with WC and VFA. *A. onderdonkii*, *B. thetaiotaomicron*, and *C. asparagiforme* were negatively correlated with WC and were also negatively correlated with FBG, HbA1c, and UC.

Clostridiales may represent potential probiotics due to their enrichment in healthy controls relative to MetS patients. A previous study investigated the use of cyclocarya paliurus polysaccharide (CCPP) to treat type 2 diabetes mellitus (Yao et al., 2020) and found that *C. asparagiforme* clearly increased with CCPP use and was accompanied by lower blood glucose levels and improvements in glucose tolerance and serum lipid parameters. These changes resulted from an increase in SCFA-producing species, which increased the level of SCFAs and upregulated SCFA-GLP1/PYY-associated sensory mediators. *C. citroniae* was also enriched in the healthy group compared with the obese cohort (Nie et al., 2020). In addition, *C. scindens* has been shown to inhibit *Clostridium difficile* infection by producing secondary bile acids that enhance resistance to *C. difficile* colonization (Mills et al., 2018). In our study, *A. onderdonkii* was found to be enriched in healthy subjects, and may therefore represent a beneficial species. Studies have previously demonstrated that species of *Alistipes* have been isolated from patients suffering from appendicitis, as well as abdominal and rectal abscesses (Parker et al., 2020). Despite this, other studies have found that the *Alistipes* species may have protective effects against some diseases, including liver fibrosis (Shao et al., 2018; Sung et al., 2019), cancer (Feng et al., 2015; Li et al., 2016), and cardiovascular disease (Jie et al., 2017; Cui et al., 2018; Jama et al., 2019). Additionally, Rau et al. (2018) showed that there was a major reduction in *A. onderdonkii* in patients with non-alcoholic fatty liver, which is consistent with our finding that there was a significant reduction in *A. onderdonkii* in MetS patients relative to the control. In addition, *B. thetaiotaomicron* (Delday et al., 2019) promoted endoscopic mucosal healing for sustained remission by reducing pro-inflammatory NF-κB signaling in these intestinal epithelial cells. *R. intestinalis* (Hiippala et al., 2018; La Rosa et al., 2019) have been shown to provide energy sources as butyrate-producing bacteria for enterocytes, which, in turn, results in anti-inflammation due to an increase in SCFA production. It is worth noting that previous studies have found that these species act as major SCFA producers, resulting in changes in host metabolism, inflammation reduction, and immune system improvements that attenuate symptoms associated with obesity and diabetes (Kumari et al., 2013; Gargari et al., 2018; Tanca et al., 2018; Gao et al., 2019).

Through functional enrichment analysis, we found that microbial pathways that were enriched in the MetS microbiome were involved in the degradation and biosynthesis of carbohydrates, fatty acids, and lipids, which may contribute to increased fermentation and utilization of SCFAs by the MetS microbiome. This, in turn, could be modulating SCFAs as a nutritional target to prevent metabolism disorders and its associated diseases such as obesity and type 2 diabetes (Hu et al., 2018). The final product of glycolysis, pyruvate, which is the metabolite of carbohydrates, fatty acids, and lipids and amino acids and participates in the TCA cycle, can be metabolized *via* different pathways, leading to the formation of (S)-lactate, formate, ethanol, or acetate, depending on the conditions, especially in P461-PWY. In the presence of excess glucose, the main fermentation product is (S)-lactate, but under glucose limitation, only formate, ethanol, and acetate are produced (Carlsson and Griffith, 1974; Yamada and Carlsson, 1975). Given that MetS is a high-sugar and high-fat state, we speculated that gut microbes may contribute to excess energy by the pathways that were responsible for the metabolism of carbohydrates and lipids. The excretion of SCFAs was increased in MetS patients (Fava et al., 2013), implying the lower level of SCFAs in the MetS patients and the higher level of SCFAs in healthy subjects, which is also consistent with our results that demonstrated that SCFA-producing microbes (*C. asparagiforme* and *R. intestinalis*) were primarily enriched in healthy subjects. In addition, we also observed that the PWY66-375 pathway resulted in inflammation by generating leukotrienes. Leukotrienes are proinflammatory metabolites of arachidonic acid that activate and amplify innate and adaptive immune responses (Murphy and Gijon, 2007; Rinaldo-Matthis and Haeggstrom, 2010). This may partially account for the increased level of inflammation we observed in MetS subjects compared to the control.

Short-chain fatty acids production through the glycolipid metabolism pathway can participate in energy metabolism in the host, decrease inflammation, and lower pathogenic bacteria colonization. Further studies investigating the targets and signaling pathways of SCFAs in the host may lead to the development of new drugs to alleviate MetS symptoms.

## Conclusion

In this study, we identified several relationships between gut microorganisms and MetS markers. WC and VFA were negatively correlated with several microorganisms and the microbiomes of MetS subjects were found to be enriched in the biosynthesis of carbohydrates, fatty acids, and lipids. These results implied that the addition of beneficial bacteria including *A. onderdonkii*, *B. thetaiotaomicron*, *C. asparagiforme*, *C. citroniae*, *C. scindens*, and *R. intestinalis* may alleviate some MetS symptoms by increasing the generation of SCFAs. The identification of specific microbial species and pathways that are closely associated with MetS might lead to new therapies for metabolic disorders.

## Data Availability Statement

The raw data supporting the conclusions of this article will be made available by the authors, without undue reservation.

## Ethics Statement

The studies involving human participants were reviewed and approved by Ethical Review Committee of Research Project of the First Affiliated Hospital of Zhengzhou University. The patients/participants provided their written informed consent to participate in this study.

## Author Contributions

QQ wrote and edited the manuscript. SY, YY, JC, and YW researched the data and performed the analysis. TL, XG, and HY helped to collect the sample and data. SW and JW were the reviewer of the work. SD was in charge of the project. All authors have read and agreed to the published version of the manuscript.

## Conflict of Interest

The authors declare that the research was conducted in the absence of any commercial or financial relationships that could be construed as a potential conflict of interest.
